# The Yin and Yang Between Plasma Glucose Levels and Cortisol Replacement Therapy in Schmidt’s Syndrome

**DOI:** 10.1177/2324709617716203

**Published:** 2017-07-06

**Authors:** Christopher A. Newton, Eleni Sheehan, Kathleen Wyne, Kenneth Cusi, Julio Leey, Hans K. Ghayee

**Affiliations:** 1 University of Florida, Gainesville, FL, USA; 2 Malcom Randall VA Medical Center, Gainesville, FL, USA; 3 The Ohio State University Wexner Medical Center, Columbus, OH, USA

**Keywords:** continuous glucose monitor, type 1 diabetes, Addison’s disease, Schmidt’s syndrome, hypoglycemia, cortisol

## Abstract

**Objective:** To illustrate how steroid replacement in adrenal insufficiency can influence the development of hypoglycemia in a patient with type 1 diabetes mellitus (T1D). **Methods:** We describe the case of a 36-year-old female patient with T1D and Addison’s disease (Schmidt’s syndrome) on multiple daily insulin injections who presented with recurrent hypoglycemia despite being on physiological replacement doses of hydrocortisone. **Results:** With the assistance of continuous glucose monitoring technology, a pattern of nocturnal hypoglycemia was clearly identified. The patient was taking her hydrocortisone 15 mg in the morning and 5 mg in the early afternoon. With the short half-life of oral hydrocortisone, the evening decline in plasma cortisol concentration led to an increased susceptibility to recurrent evening and nocturnal hypoglycemia. Hypoglycemic episodes were resolved when her morning hydrocortisone dose was changed and prednisolone was added to a later time in the evening. **Conclusion:** Patients with Schmidt’s syndrome can be susceptible to nocturnal hypoglycemia with inadequate steroid replacement. Identifying patients at risk for hypoglycemia in Schmidt’s syndrome provides an opportunity for precision management beyond the manipulation of antihyperglycemic agents.

## Introduction

Schmidt’s syndrome, also known as polyglandular syndrome type II, is defined as an autoimmune disease of the adrenal glands, pancreatic islets, thyroid gland, and, very rarely, the parathyroid glands.^1^ Hypoglycemia is an important challenge in patients with type 1 diabetes mellitus (T1D). Many patients have chronic autonomic neuropathy with hypoglycemia unawareness and an inadequate glucagon response that make them uniquely prone to severe hypoglycemia. Patients with Schmidt’s syndrome are at greater risk of hypoglycemia by having primary adrenal insufficiency (Addison’s disease). It is estimated that the prevalence of Addison’s disease in T1D is 0.3%.^2^ Therefore, glucocorticoid replacement must be carefully tailored and monitored closely to minimize hypoglycemic episodes and use insulin therapy safely. In this setting, diabetes control and adrenal glucocorticoid replacement turns into a difficult balancing act. Patients with Addison’s disease may experience hypoglycemia if their replacement dose is not high enough.^3^ However, there is limited guidance in the literature as to how to manage such patients and which is the best approach to prevent hypoglycemia in this population.

In order to unravel the etiology of hypoglycemia in patients with Schmidt’s syndrome, physicians need to perform a thorough history and should consider utilizing new advances in technology, such as continuous glucose monitoring (CGM) systems. However, although rare reports of hypoglycemia in Addison’s disease have been reported in the literature,^4,5^ diagnosis of hypoglycemia using CGM in patients with Schmidt’s syndrome has not been previously described. This is unfortunate at a time when the severe consequences of recurrent hypoglycemia are being increasingly recognized in patients with T1D.^5-7^ Within this context, the current report makes a compelling case for health care providers to consider underlying causes of severe recurrent hypoglycemia in patients with autoimmune syndromes.

## Case

A 36-year-old woman with T1D for 24 years presented for an unscheduled appointment requesting to discuss evening and early morning hypoglycemia. A few times in the recent past, her husband found her unresponsive in the middle of the night with hypoglycemia and administered glucagon. Her diabetes medication regimen was basal insulin glargine (Lantus) 18 units at 2 pm and rapid-acting premeal insulin glulisine (Apidra). The patient used 1 unit of insulin per 10 grams of carbohydrates and a correction factor of 1 unit of insulin per 100 mg/dL of glucose above the normal range to control her blood sugars. Given that she was having frequent hypoglycemic episodes, mainly in the evening and during the night, she had actually stopped her prandial insulin and was only using the rapid-acting insulin for hyperglycemia if the plasma glucose concentration was ≥300 mg/dL. She also reported frequent early morning hypoglycemia (4-7 am), some of which were followed by an episode of hyperglycemia that she would then treat with 2 units of insulin glulisine if glucose was >300 mg/dL. Despite stopping her prandial insulin and gradually lowering her basal insulin dose, she was unable to control her plasma glucose levels and had grown increasingly fearful that she would not awaken from a low blood sugar during the night. The patient denied any injection site issues.

To compound her high risk of severe hypoglycemia, the patient had hypoglycemia unawareness from autonomic neuropathy. She reported that she would have symptoms of hypoglycemia only when her blood glucose would become ≤20 to 40 mg/dL. She was taking hydrocortisone 15 mg every morning at 8 am and 5 mg at 3 pm as replacement therapy for Addison’s disease. She had poor compliance taking her fludrocortisone.

**Figure 1.**
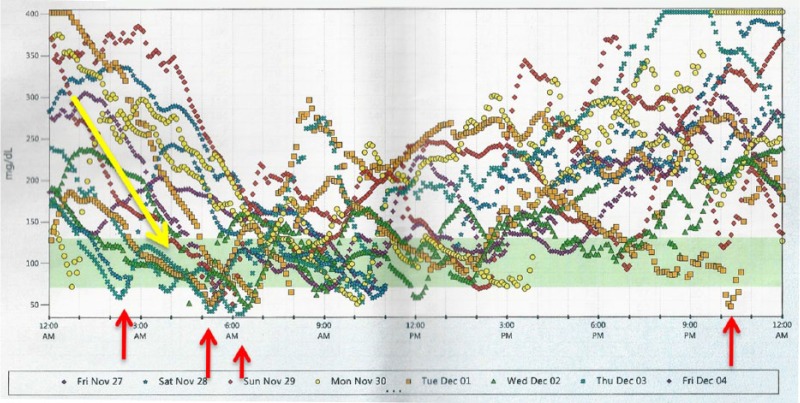
Continuous glucose monitoring (CGM; Dexcom) with red arrows demonstrating nocturnal hypoglycemia between midnight and 6 am. The yellow arrow indicates an overall downward nocturnal plasma glucose level trend.

Her physical exam revealed peripheral neuropathy involving her lower extremities. Vital signs and blood glucose concentrations were normal during the initial clinic visit. Her CGM is shown in Figure 1 and is characterized by a pattern of recurrent nocturnal hypoglycemia. It was suspected that she was receiving an adequate amount of basal and premeal insulin but that the most likely cause of late evening and early morning hypoglycemia was an insufficient plasma cortisol concentration between midnight and 6 am. She denied use of paracetamol/acetaminophen, which could have interfered with the CGM readings.

Her laboratory values are listed in Table 1. Briefly, routine chemistries, plasma thyroid function, and IGF-1 concentrations were within normal limits. However, she did have a low morning plasma cortisol level. Based on these findings, the insulin glargine dose was decreased slightly from 18 to 16 units, and more importantly, her cortisol replacement regimen was changed to hydrocortisone 20 mg at 9 am and prednisolone 3 mg added with dinner in the evenings. The reason why prednisolone at 3 mg was used is because it has a longer biological half-life compared with hydrocortisone. This dose of prednisolone is equivalent to hydrocortisone 12 mg, thereby reducing any chances of nocturnal hypoglycemia. Soon after this change was instituted, her hypoglycemia significantly improved.

**Table 1.** Laboratory Results.

	Laboratory Value	Reference Range	Date
AM cortisol (µg/dL)	1.9	6.2-19.4	October 9, 2013, 07:50 am
AM cortisol (µg/dL)	14.0	6.2-19.4	January 28, 2016, 09:55 am (after taking prednisolone in evening)
IGF-1 (ng/mL)	119	106-277	January 28, 2016, 09:55 am
TSH (mIU/mL)	0.85	0.27-4.20	November 1, 2015, 14:00 pm

Abbreviation: TSH, thyroid-stimulating hormone.

## Discussion

One of the biggest challenges in the management of diabetes is detecting nocturnal hypoglycemia in susceptible patients.^6^ Patients with T1D are aware of the high mortality rates once autonomic neuropathy and hypoglycemia unawareness have developed. Mechanistically, low glucose can lead to cardiac arrhythmias by prolonging the Q-T interval.^7,8^ The sympathetic nervous system is activated in hypoglycemia in order to raise plasma glucose concentrations by raising epinephrine levels. However, episodes of hypoglycemia can ameliorate the sympathoadrenal response to subsequent hypoglycemic events.^9^

The adrenal cortex supplies the medulla with cortisol via portal venous system where it helps catalyze the conversion from norepinephrine to epinephrine through the enzyme phenylethanolamine-N-mehtyltransferase (PNMT).^10^ Hence, with Addison’s disease, where the adrenal gland is nonfunctional, epinephrine deficiency will also be present. In scenarios where a patient has hypoglycemia and primary adrenal insufficiency, the patient would have a reduced epinephrine response to hypoglycemia.^11^ Inadequate plasma cortisol and epinephrine concentrations increased our patient’s risk of developing hypoglycemia. In addition, her high risk of hypoglycemia was compounded by long-standing diabetes with inadequate glucagon secretion and autonomic neuropathy with hypoglycemia unawareness. This effect was exacerbated by frequent hypoglycemic events.^12^ Once nocturnal hypoglycemia was documented by CGM, it resolved when her hydrocortisone dose was adjusted from an early afternoon administration to prednisolone in the evening hours. However, clinicians must also weigh the balance between resolving any future hypoglycemic episodes with the steroid excess causing metabolic changes such as weight gain, hypertension, and osteoporosis.

Other differential diagnosis for patients with hypoglycemia include excessive insulin dosing, pronounced peak of basal insulin, and growth hormone deficiency. IGF-1 levels were within normal range in our patient. When the patient presented to clinic with complaints of nocturnal hypoglycemia along with the information regarding her steroid regimen, a hypothesis of inadequate nocturnal cortisol levels was born. This hypothesis was further supported by CGM findings. The clinical response the patient had as a result of changes in her steroid timing was suggestive of this hypothesis being valid. Although the dosing of insulin glargine was reduced by 2 units, this slight change was less likely to be as significant as the change in steroids, especially since the patient experimented with lower glargine doses in the past along with stopping prandial insulin.

This case illustrates how inadequate steroid replacement may predispose to nocturnal hypoglycemia. Understanding this ying and yang of glucose and cortisol metabolism led to the resolution of our patient’s frequent nocturnal hypoglycemia and can radically improve the quality of life of a patient.
